# Supplementary material for: Combining evidence from Mendelian randomization and colocalization: Review and comparison of approaches
Source: Am J Hum Genet. Author manuscript; Available in PMC 2022 May 17. (PMC7612737; doi:10.1016/j.ajhg.2022.04.001)
Supplement: Supplementary File 1 [file EMS144703-supplement-Supplementary_File_1.pdf]

The American Journal of Human Genetics, Volume *109*

Supplemental information

Combining evidence from Mendelian randomization and colocalization: Review and comparison of approaches

Verena Zuber, Nastasiya F. Grinberg, Dipender Gill, Ichcha Manipur, Eric A.W. Slob, Ashish Patel, Chris Wallace, and Stephen Burgess

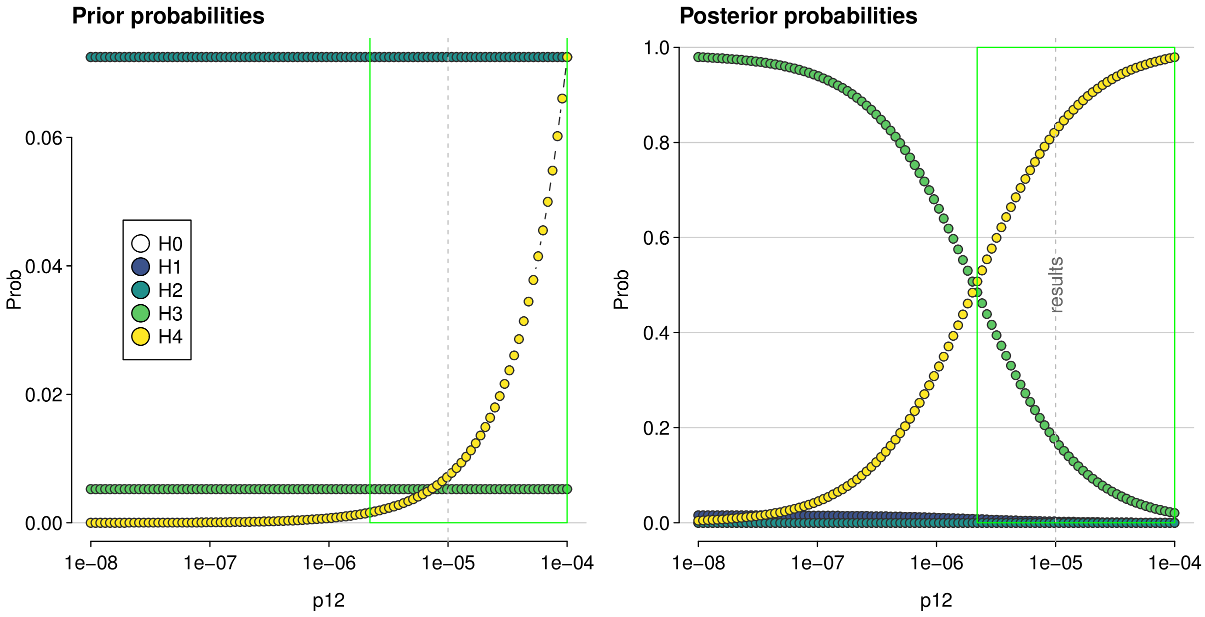

Figure S1: Sensitivity analysis varying the prior p_{12}, which represents the prior probability that any single variant is a causal variant for both traits. The left panel displays the prior probabilities of the hypotheses, the right panel displays the posterior probabilities. For low values of p_{12}, the method prefers the $\mathbb{H}_3$ hypothesis (distinct causal variants), whereas for greater values of p_{12}, the method prefers the $\mathbb{H}_4$ hypothesis (shared causal variant).
