# Supplementary material for: Combining evidence from Mendelian randomization and colocalization: Review and comparison of approaches
Source: Am J Hum Genet. Author manuscript; Available in PMC 2022 May 17. (PMC7612737; doi:10.1016/j.ajhg.2022.04.001)
Supplement: Supplementary File 2 [file EMS144703-supplement-Supplementary_File_2.pdf]

Please cite this article in press as: Zuber et al., Combining evidence from Mendelian randomization and colocalization: Review and comparison of approaches, The American Journal of Human Genetics (2022), https://doi.org/10.1016/j.ajhg.2022.04.001

REVIEW

Combining evidence from Mendelian randomization and colocalization: Review and comparison of approaches

Verena Zuber,[1,2,3,12] Nastasiya F. Grinberg,[4,12] Dipender Gill,[1,5,6,7] Ichcha Manipur,[8,9] Eric A.W. Slob,[10] Ashish Patel,[10] Chris Wallace,[8,9,10] and Stephen Burgess[10,11,*]

Summary

Mendelian randomization and colocalization are two statistical approaches that can be applied to summarized data from genome-wide association studies (GWASs) to understand relationships between traits and diseases. However, despite similarities in scope, they are different in their objectives, implementation, and interpretation, in part because they were developed to serve different scientific communities. Mendelian randomization assesses whether genetic predictors of an exposure are associated with the outcome and interprets an association as evidence that the exposure has a causal effect on the outcome, whereas colocalization assesses whether two traits are affected by the same or distinct causal variants. When considering genetic variants in a single genetic region, both approaches can be performed. While a positive colocalization finding typically implies a non-zero Mendelian randomization estimate, the reverse is not generally true: there are several scenarios which would lead to a non-zero Mendelian randomization estimate but lack evidence for colocalization. These include the existence of distinct but correlated causal variants for the exposure and outcome, which would violate the Mendelian randomization assumptions, and a lack of strong associations with the outcome. As colocalization was developed in the GWAS tradition, typically evidence for colocalization is concluded only when there is strong evidence for associations with both traits. In contrast, a non-zero estimate from Mendelian randomization can be obtained despite only nominally significant genetic associations with the outcome at the locus. In this review, we discuss how the two approaches can provide complementary information on potential therapeutic targets.

Introduction

Genome-wide association studies (GWASs) have been fruitful in identifying genetic variants that are related to various traits and diseases. They also provide a rich source of data that can be leveraged in downstream analyses to better understand biological mechanisms linking traits and diseases.[1,2] Two statistical approaches routinely used in “post-GWAS” analyses are Mendelian randomization and colocalization. Mendelian randomization takes an exposure and an outcome and uses genetic variants to provide evidence supporting or refuting the hypothesis that the exposure has a causal effect on the outcome. Colocalization takes two traits and considers whether their genetic associations at a locus are explained by overlapping or distinct variants. Although the two approaches were developed separately in different scientific communities, there are similarities between the approaches in both their objectives and practice.

In this review, we introduce the two approaches before contrasting them in terms of their assumptions, application, and interpretation. We then explain why the two approaches may give apparently conflicting results and how this can be resolved through understanding the different viewpoints of the methods. We provide some examples where the approaches provide complementary information on causal pathways and potential therapeutic targets, considering the relationships of low-density lipoprotein (LDL) cholesterol with coronary heart disease (CHD) and with Alzheimer’s disease. We compare results from polygenic Mendelian randomization analyses to those from Mendelian randomization and colocalization for variants in the *PCSK9* and *APOE* regions. Finally, we discuss future directions for the methods and offer recommendations on use of the approaches in practice to address distinct but related questions.

Mendelian randomization

Conceptual overview

It is well known that “correlation is not causation.” An observational association between a risk factor (which we refer to as the exposure) and an outcome may arise for several reasons, including confounding and reverse

[1]Department of Epidemiology and Biostatistics, School of Public Health, Imperial College London, London, UK; [2]MRC Centre for Environment and Health, School of Public Health, Imperial College London, London, UK; [3]UK Dementia Research Institute at Imperial College, Imperial College London, London, UK; [4]National Institute of Agricultural Botany, Cambridge, UK; [5]Clinical Pharmacology and Therapeutics Section, Institute of Medical and Biomedical Education and Institute for Infection and Immunity, St George’s, University of London, London, UK; [6]Clinical Pharmacology Group, Pharmacy and Medicines Directorate, St George’s University Hospitals NHS Foundation Trust, London, UK; [7]Genetics Department, Novo Nordisk Research Centre Oxford, Oxford, UK; [8]Cambridge Institute of Therapeutic Immunology & Infectious Disease, University of Cambridge, Cambridge, UK; [9]Department of Medicine, School of Clinical Medicine, University of Cambridge, Cambridge, UK; [10]MRC Biostatistics Unit, University of Cambridge, Cambridge, UK; [11]Cardiovascular Epidemiology Unit, Department of Public Health and Primary Care, University of Cambridge, Cambridge, UK
[12]These authors contributed equally
*Correspondence: sb452@medschl.cam.ac.uk
https://doi.org/10.1016/j.ajhg.2022.04.001.
© 2022 American Society of Human Genetics.

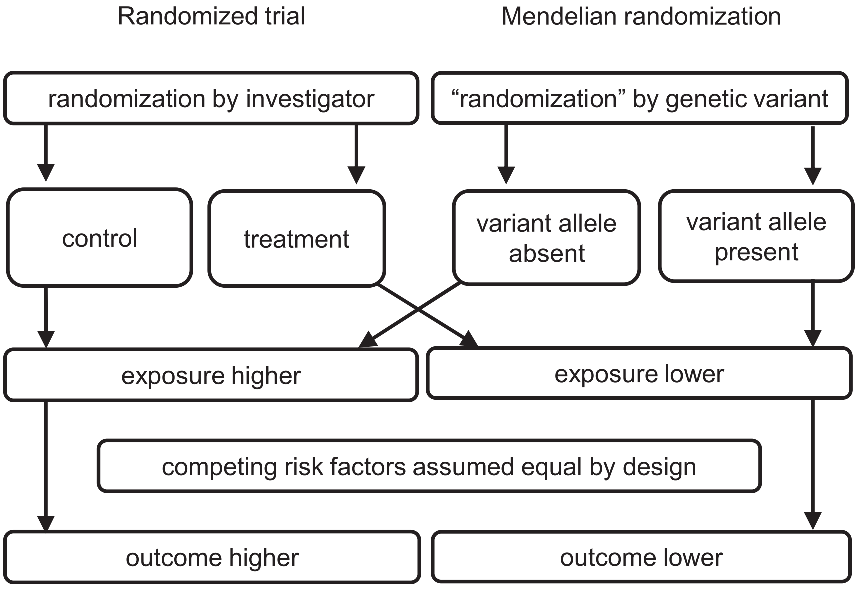

Figure 1. Schematic diagram illustrating analogy between Mendelian randomization and randomized trial
Adapted from Hingorani and Humphries.[4]

causation. The ideal way to detect a causal effect of an exposure on an outcome is a randomized controlled trial, in which the population is randomly divided into groups that are given different treatment regimens—typically, in the treatment group there is an intervention on the exposure (such as LDL-cholesterol lowering medication if the exposure is LDL cholesterol) and in the control group there is no intervention. Provided the groups are otherwise treated identically, any association between treatment assignment and the outcome must be attributed to a causal effect of the exposure.[3] In Mendelian randomization, rather than individuals being randomly assigned to different regimens by an investigator, we assume that specifically chosen genetic variants behave analogously to treatment assignment, dividing the population into subgroups in a way that mimics randomization (Figure 1).[4,5] In order to obtain valid causal inferences for the effect of the exposure, variants must (1) divide the population into subgroups with different average levels of the exposure, but (2) not be associated with the outcome via confounding pathways, and (3) not influence the outcome directly, but only potentially indirectly via the exposure.[6] These three assumptions (referred to as relevance, exchangeability, and exclusion restriction, respectively) form the definition of an instrumental variable.[7]

Genetic variants are plausible candidate instrumental variables for several reasons: they may regulate a gene that has a specific effect on the exposure of interest; genetic variants are inherited at random conditional on parental genotypes (following Mendel's law of independent assortment),[8] implying that variants should typically not be associated with traits that represent competing risk factors, as has been observed in empirical investigations;[9,10] and the genotype is generally fixed from conception, meaning that it cannot be influenced by environmental confounders, and further providing protection against reverse causation.[11] However, the primary motivation of Mendelian randomization is epidemiological rather than genetic. Genetic variants are tools to assess causal relationships, rather than being the primary focus of interest.

Different versions of Mendelian randomization

There are several ways that Mendelian randomization analyses can be implemented, depending on the exposure under investigation and the data being analyzed. When the technique was first proposed, analyses typically used individual-participant data on the genetic variants, exposure, and outcome in the same dataset ("one-sample Mendelian randomization").[12] However, the popularity of the approach has risen sharply with two innovations: first, methods for performing Mendelian randomization analyses using summarized data, namely beta-coefficients representing the estimated marginal genetic associations with the exposure and outcome;[13] and second, "two-sample Mendelian randomization," in which genetic association estimates are obtained from one dataset for the exposure and from a second dataset for the outcome.[14] This is often for pragmatic reasons, as genetic associations with exposures are typically estimated in cross-sectional studies, whereas genetic associations with outcomes are typically estimated in longitudinal or case-control studies. These two innovations are regularly applied together when Mendelian randomization is implemented using publicly available summarized genetic association data from GWAS investigations.[15]

A further important distinction is between Mendelian randomization analyses using variants from a single gene region (*cis*-Mendelian randomization) and those using variants from multiple gene regions (polygenic Mendelian randomization).[16] The former is common when analyzing an exposure that is a gene product (such as mRNA expression or circulating levels of a protein); the latter is common when the exposure is a complex multifactorial trait (such as blood pressure or body mass index). Several methods have been developed for polygenic Mendelian randomization that are robust to violations of the instrumental variable assumptions for some of the genetic variants.[17] However, while some robust approaches have been proposed for *cis*-Mendelian randomization,[18,19] they cannot be applied uniformly to all analyses. As most drug targets are proteins, Mendelian randomization analyses for pharmacological target validation are typically *cis*-Mendelian randomization analyses.[20,21]

Please cite this article in press as: Zuber et al., Combining evidence from Mendelian randomization and colocalization: Review and comparison of approaches, The American Journal of Human Genetics (2022), https://doi.org/10.1016/j.ajhg.2022.04.001

Colocalization

Conceptual overview

Neighboring genetic variants tend to be inherited together and hence are typically correlated, a phenomenon known as linkage disequilibrium. Given the hundreds of thousands of genetic associations identified to date, one concern is that two traits may be causally influenced by distinct variants that happen to be correlated with each other. This could lead to violation of the Mendelian randomization assumption of exchangeability by providing a pathway between a genetic variant and the outcome that does not pass through the exposure. For instance, a genetic predictor of the exposure could be in linkage disequilibrium with another variant that independently influences the outcome, either directly or via an alternative risk factor. Separately from the development of Mendelian randomization by epidemiologists, researchers in GWASs were concerned that associations between variants and disease endpoints had limited mechanistic interpretation, and so developed colocalization methods to assess whether disease endpoints and potential biological mediators might share one or more causal variants.[22–24]

We define a causal variant as a genetic polymorphism for which variation in the genotype directly impacts molecular mechanisms that have a consequent effect on the trait of interest. Changing the genotype at this position (for instance, using gene editing technology[25]) would lead to changes in downstream variables. This is in contrast to a tagging variant, which is correlated with a causal variant through linkage disequilibrium, though it has no direct effects on the trait of interest. Colocalization attempts to discern between two possible underlying situations at a genetic region (Figure 2, top row): distinct causal variants, possibly in linkage disequilibrium, or a single shared signal (colocalization). Colocalization can be viewed as an extension of fine-mapping to multiple traits. The goal of fine-mapping is to detect the causal variants for a single trait, with the subsequent aim to understand the biological relevance of such variants.[26] Colocalization considers the overlap between causal variants for two (or more[27,28]) traits.

Colocalization is increasingly being used as part of Mendelian randomization investigations to assess the instrumental variable assumptions for a given genetic region. If there is strong evidence that the exposure and outcome are influenced by distinct causal variants, then it is implausible that variants in that region are valid instrumental variables for the exposure.

Different versions of colocalization

Two families of methodological approaches that have been developed for colocalization are proportional colocalization and enumeration colocalization. In proportional colocalization, the null hypothesis is proportionality of the genetic associations with the two traits.[22] If there is colocalization, we would expect marginal genetic associations with the two traits to be proportional provided that either there is a single causal variant (in which case the genetic associations would be determined by their correlation with the causal variant) or the traits are on the same causal pathway and all variants primarily influence the same upstream trait (which may be one of the traits under analysis, or an unmeasured trait). If there is evidence against the statistical model that the genetic associations are proportional, then we conclude that there is lack of colocalization. Otherwise, we conclude that there is colocalization.

In enumeration colocalization, the analyst compares evidence for different hypotheses in a Bayesian framework. An example of a method in this family is the coloc method.[24] For simplicity, we suppose there are two traits and work under the assumption that there is at most one causal variant per trait. The hypotheses are: $\mathbb{H}_0$, no association with either trait; $\mathbb{H}_1$: association with trait 1 but not trait 2; $\mathbb{H}_2$, association with trait 2 but not trait 1; $\mathbb{H}_3$, association with both traits but at separate causal variants; and $\mathbb{H}_4$, association with both traits at a shared causal variant.[24] Of these, hypotheses $\mathbb{H}_3$ and $\mathbb{H}_4$ are of the most interest, with $\mathbb{H}_4$ corresponding to colocalization. The posterior probability for each of these hypotheses can be calculated from the prior probability (which is set by the investigator) and summarized genetic association data, which are used to compute approximate Bayes factors that represent the contribution from the likelihood.[29] A conceptual advantage of enumeration colocalization is that the method only concludes that there is colocalization in the presence of positive evidence supporting colocalization. In the absence of evidence, posterior probabilities will approximate the prior probabilities, which can therefore be set to avoid spurious results.

Other colocalization methods align broadly with one or other of these families of approaches. For example, the heterogeneity in dependent instruments (HEIDI) test[30] assesses heterogeneity in genetic associations, and so falls into the proportional colocalization family. The expression quantitative trait locus (eQTL) Causal Variant Identification in Associated Regions (eCAVIAR) method[31] performs fine-mapping for two traits simultaneously allowing the possibility of multiple causal variants per trait, and thus falls in the enumeration family of methods. However, the eCAVIAR method provides findings on a variant level rather than a regional level, and so results from this method are less directly comparable to those from Mendelian randomization; hence we do not consider this method further in this review.

While findings from coloc are fairly robust to violations of the assumption of a single causal variant,[24] enumeration colocalization methods have been developed that relax this assumption. The original solution was to perform stepwise regression on each trait and to perform colocalization for each of the pairs of signals.[24] However, given the high degree of correlation between genetic variants, stepwise regression can fail to identify the true causal variants.[32] Various Bayesian fine-mapping methods have been developed for GWAS summary statistics which outperform

Please cite this article in press as: Zuber et al., Combining evidence from Mendelian randomization and colocalization: Review and comparison of approaches, The American Journal of Human Genetics (2022), https://doi.org/10.1016/j.ajhg.2022.04.001

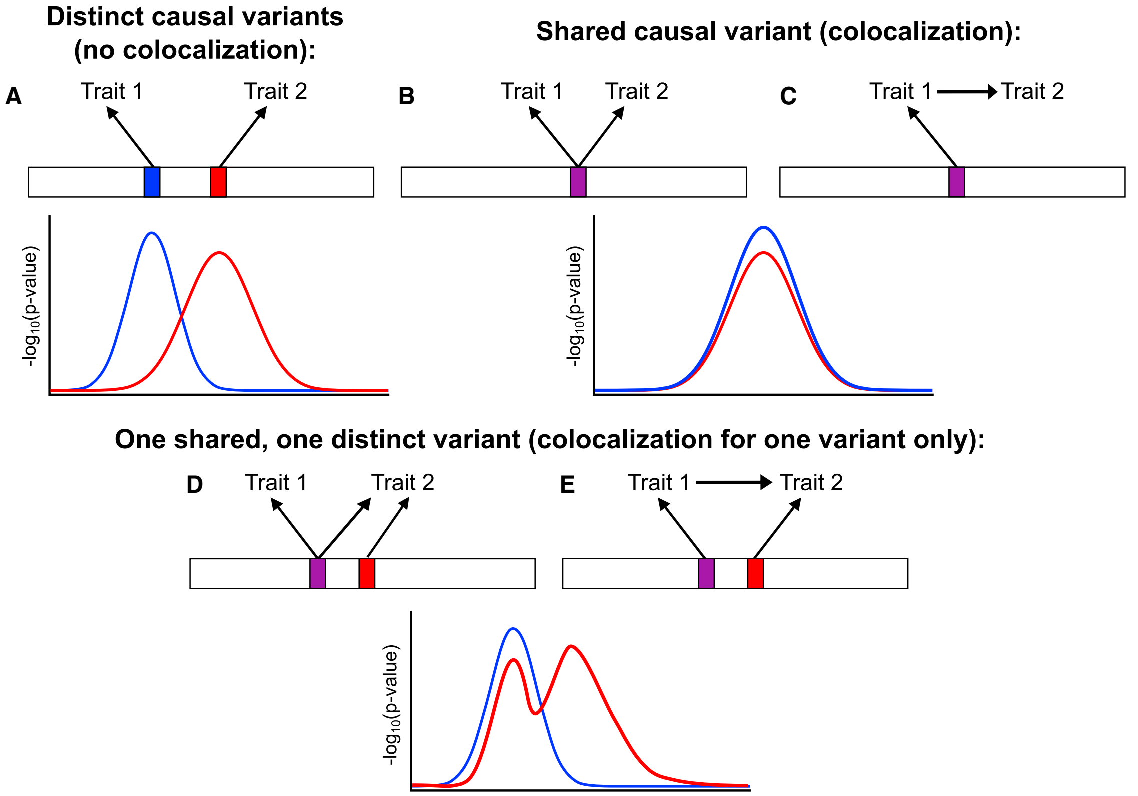

Figure 2. Schematic diagrams illustrating colocalization in five scenarios
(A) Two traits with distinct causal variants in linkage disequilibrium.
(B) Two unrelated traits with a shared causal variant.
(C) Two traits with a shared causal variant where the first trait influences the second trait.
(D and E) One shared causal variant and one distinct causal variant for trait 2.
Scenarios (B) and (C) are examples of colocalization. For scenarios (D) and (E), there is colocalization at the shared variant, but not at the distinct variant. Colocalization is unable to distinguish between the scenarios in which trait 1 and trait 2 are causally unrelated (scenarios B and D), and in which trait 1 has a causal effect on trait 2 (scenarios C and E). Illustrative regional association plots for each scenario represent the negative $\log_{10}$ p values for associations of variants with each trait (blue for trait 1, red for trait 2) plotted against chromosomal position.

stepwise regression. In the context of colocalization, the coloc-Sum of Single Effects (SuSiE) method first performs fine-mapping for each trait using the SuSiE method to decompose genetic associations into a sum of single causal variant summary statistics, each of which are then analyzed in parallel using the coloc method.[33] The SuSiE method fits a sparse model for each trait in terms of the genetic variants as a sum of vectors, each of which has only one non-zero component.[34,35] The colocalization and fine-mapping in the presence of allelic heterogeneity (CAFEH) method fits a hierarchical Bayesian model that implements colocalization by performing simultaneous fine-mapping for multiple traits.[36] Both approaches should provide more reliable colocalization inferences when there are multiple causal variants in one genetic region, including the potential scenario that colocalization is detected for one causal variant, but not at another (Figure 2, bottom row). In such a scenario, enumeration colocalization methods that assume a single causal variant would typically conclude in favor of colocalization, whereas proportional colocalization methods would typically conclude that there is a failure to colocalize.

Comparison between Mendelian randomization and colocalization

Similarity of statistical analysis model

While there are many methods for both Mendelian randomization and colocalization, the inverse-variance weighted (IVW) method that is typically used as the primary analysis method for Mendelian randomization and the proportional colocalization method have several similarities. We index genetic variants by j, denoting the estimated genetic association with the exposure for variant j as $\widehat{\beta}_{Xj}$ and its standard error as $\mathrm{se}(\widehat{\beta}_{Xj})$, and the estimated genetic association with the outcome as $\widehat{\beta}_{Yj}$ and its standard error as $\mathrm{se}(\widehat{\beta}_{Yj})$. The IVW method is equivalent to fitting the following regression model:

$$\widehat{\beta}_{Yj} = \theta\,\widehat{\beta}_{Xj} + \varepsilon_j, \quad \varepsilon_j \sim \mathcal{N}\left(0, \mathrm{se}\left(\widehat{\beta}_{Yj}\right)^2\right), \qquad \text{(Equation 1)}$$

where θ is the causal parameter.[37] The regression model is a straight line through the origin with slope θ. The variance of the error term ε_j is potentially different for each genetic

variant, depending on the precision of the genetic association with the outcome, which in turn depends on the sample size and minor allele frequency for that variant. A random-effects version of the IVW method can be obtained by additionally estimating a heterogeneity parameter φ, which represents the residual standard error in the regression model:

$$\widehat{\beta}_{Yj} = \theta\, \widehat{\beta}_{Xj} + \varepsilon_j, \quad \varepsilon_j \sim \mathcal{N}\left(0, \varphi^2 \mathrm{se}\left(\widehat{\beta}_{Yj}\right)^2\right). \qquad \text{(Equation 2)}$$

This parameter is allowed to take values 1 or more. Larger values represent overdispersion, meaning that the $\widehat{\beta}_{Yj}$ estimates are more variable than would be expected due to chance alone.[38] Values of φ below one would represent underdispersion, meaning less variability than expected by chance alone, which is not logically plausible. If genetic variants are correlated, then the separate error terms ε_j should be replaced by an error vector that takes a multivariable normal distribution, requiring generalized weighted linear regression to obtain the IVW estimate.[37]

Proportional colocalization fits an equivalent model under the null hypothesis (using the same notation) that there exists a parameter θ such that $\beta_{Yj} = \theta\beta_{Xj}$ for all j, where the absence of hats indicates these are the genetic associations, not their estimates. However, rather than focusing on the slope parameter θ, colocalization treats this as a nuisance parameter and focuses on the distribution of the error terms. Wallace et al.[39] showed that a valid test statistic (equivalent to the test statistic of Plagnol et al.[22]) can be calculated by taking the vectors of estimated associations $\widehat{\boldsymbol{\beta}}_{\mathbf{X}}$ and $\widehat{\boldsymbol{\beta}}_{\mathbf{Y}}$, which are assumed to follow multivariate normal distributions:

$$\widehat{\boldsymbol{\beta}}_{\mathbf{X}} \sim \mathcal{N}(\boldsymbol{\beta}_{\mathbf{X}}, V_X), \quad \widehat{\boldsymbol{\beta}}_{\mathbf{Y}} \sim \mathcal{N}(\boldsymbol{\beta}_{\mathbf{Y}}, V_Y), \qquad \text{(Equation 3)}$$

where bold face denotes a vector, $\mathrm{cov}(\widehat{\boldsymbol{\beta}}_{\mathbf{X}}, \widehat{\boldsymbol{\beta}}_{\mathbf{Y}}) = 0$ (equivalent to a two-sample assumption in Mendelian randomization), and the variance-covariance matrices V_X and V_Y are assumed to be known. Then, if we define $\mathbf{u} = \widehat{\boldsymbol{\beta}}_{\mathbf{X}} - \frac{1}{\theta}\, \widehat{\boldsymbol{\beta}}_{\mathbf{Y}}$ and $V = V_X + \frac{1}{\theta^2} V_Y$, the test statistic:

$$T(\theta) = \mathbf{u}^T\, V^{-1}\, \mathbf{u} \qquad \text{(Equation 4)}$$

has a chi-squared distribution on p degrees of freedom under the null hypothesis, where p is the number of genetic variants. As the slope parameter θ is unknown, its maximum likelihood estimate can be substituted to obtain a test statistic $T(\widehat{\theta})$ on $p - 1$ degrees of freedom. This test statistic is similar to Cochran's Q heterogeneity statistic to compare the variant-specific estimates (i.e., $\frac{\widehat{\beta}_{Yj}}{\widehat{\beta}_{Xj}}$ for each variant j) that is estimated in Mendelian randomization analyses,[40] and the HEIDI test statistic. The major difference is that Cochran's Q heterogeneity statistic typically uses the IVW estimate for the slope parameter, not its maximum likelihood estimate.

Hence, although the approaches of Mendelian randomization and colocalization are different, they can be implemented using the same statistical model, but Mendelian randomization focuses on the slope whereas proportional colocalization focuses on the variability of errors in the model. The goal of proportional colocalization is to establish whether a line anchored at the origin can be drawn through the genetic associations, whereas Mendelian randomization estimates the slope of the line assuming that such a line exists.

While these similarities are instructive to compare the Mendelian randomization and colocalization approaches, proportional colocalization has fallen out of use due to a number of practical problems. One is that the null hypothesis is the presence of colocalization. This means that the analyst is searching for evidence against colocalization, and concludes that there is colocalization in the absence of evidence to the contrary. If there is little information in the data, it may be difficult to detect evidence against colocalization. A related problem is that of selecting variants for the test. Genetic regions contain hundreds of variants, of which only a subset will truly causal affect either trait, and therefore be informative. If we use all variants in the region, the test will have limited power. If we select some subset at random, we are likely to limit power by discarding informative variants. If instead we select a subset based on strength of association with one or both traits, the association estimates are biased because they have been selected on the basis of their extreme values, and so the type 1 error control of the test is lost, perhaps spectacularly.[23] Mendelian randomization estimates are also affected by this selection bias, known as winner's curse,[41] which is closely related to weak instrument bias.[42] Such bias is typically minimal when genetic variants are associated with the exposure at a genome-wide level of significance, and type 1 error inflation can only occur when genetic associations with the exposure and outcome are estimated in overlapping samples.[43]

Given these difficulties, in the remainder of this review we will focus on the more widely used enumeration colocalization approach, which does not require selection of genetic variants for a given region of interest.

Conceptual and practical differences

While there are clear similarities between Mendelian randomization and colocalization (both approaches use genetic variants to elucidate the nature of the relationship between traits), there are also conceptual and practical differences.

The motivation for Mendelian randomization is the existence of an exposure variable that is a candidate causal risk factor, and the objective is to assess evidence for a potential causal effect of the exposure on an outcome. Hence the choice of genetic variants in the analysis is determined by their associations with the exposure. As the analysis assesses whether the variants are associated with the outcome or not, variants are included in the analysis regardless of their association with the outcome. The motivation for a colocalization analysis is a section of the

genome, usually a gene region, where there are probable signals for association with both of the traits of interest, and the objective is to determine whether these signals are driven by the same variants.

In a polygenic Mendelian randomization analysis, it is typical to include one variant in the analysis per genetic region, although precision of the estimate can be improved if there are multiple variants in the genetic region that explain independent variance in the exposure (i.e., conditionally independent hits).[37] However, it is uncommon to find more than a few variants per region that explain a substantial fraction of variance in the exposure. Additionally, when using robust methods for Mendelian randomization, it may be preferable to include one variant per region to ensure that the analysis is not too dependent on the validity of genetic variants from a single region.[16] A *cis*-Mendelian randomization analysis can be performed using a single variant or multiple variants in the same genetic region that are conditionally independent predictors of the exposure. A colocalization analysis typically includes as many variants as are available in the genetic region surrounding the lead variant. This is because the method assumes that the causal variant is within the set of variants studied, though because of linkage disequilibrium the method is fairly robust as long as a dense map of variants is available. In contrast, it is not necessary for variants used in Mendelian randomization to be causal variants for the exposure, as all that is needed is for the variants to divide the population into subgroups with different average levels of the exposure. This is because the method assesses the causal nature of the exposure, not of the genetic variants. However, non-causal “tagging” variants may be more likely to violate the exclusion restriction assumption due to pleiotropic effects on other causal pathways.

In Mendelian randomization, one of the traits is the exposure trait and the other is the outcome trait, whereas in colocalization the traits are treated symmetrically in the analysis. Mendelian randomization analyses can be performed in two directions (known as bidirectional Mendelian randomization); considering whether genetic predictors of the exposure are associated with the outcome, and considering whether genetic predictors of the outcome are associated with the exposure (representing causation in the opposite direction[44]).

Genetic variants used in a Mendelian randomization analysis are assumed to follow the assumptions of an instrumental variable. In particular, they are assumed to be specifically related with the exposure and not associated with any other traits unless those traits are downstream consequences of the exposure. These assumptions imply that an association of the variant with the outcome can only arise due to a causal effect of the exposure on the outcome.[6] In contrast, no such assumption is made in colocalization.

The output from a Mendelian randomization analysis is an estimate, representing the association of genetically predicted levels of the exposure with the outcome.[45] This provides evidence on the strength of the causal effect of the exposure on the outcome, as well as its direction. Under the assumption that differences in the exposure between genetically defined subgroups of the population can be replicated by a clinical intervention on the exposure, as well as technical assumptions such as linearity of the causal effect of the exposure on the outcome,[46] the Mendelian randomization estimate represents the causal effect of the exposure on the outcome. However, there are many reasons why genetic differences in the exposure qualitatively differ from clinical interventions on an exposure in practice.[47,48] For example, genetic differences are typically small but life-long, whereas clinical interventions are typically larger in magnitude of change in the exposure, but applied later in life. Hence, some authors have advocated either not presenting causal estimates, or the primary interpretation of a causal estimate being a test of a causal hypothesis, rather than an estimate of a causal effect.[45,49,50] The output from an enumeration colocalization analysis is a set of Bayesian posterior probabilities, with different posterior probabilities representing the strength of evidence for the competing hypotheses.

Differences in interpretation of results

There are also differences between the approaches in their interpretation of results. Compared with GWAS investigations, *cis*-Mendelian randomization analyses have a relatively low evidential threshold for providing a non-zero estimate; they can provide a non-zero estimate even if none of the variants are strongly associated with the outcome. This is justified by a strong prior belief that the genetic variant(s) in the analysis can be interpreted as proxies for intervention on the exposure. In a polygenic Mendelian randomization analysis, primary evidence for a non-zero estimate is less important than consistency of the evidence across genetic variants, which can be assessed using robust methods. A causal claim is more reasonable if genetic variants across different gene regions are concordantly associated with the outcome.[51] In contrast, while colocalization analyses would typically not be attempted unless there was some statistical evidence for an association with the at least one trait in the genetic region, strong evidence of associations with both traits are required to support colocalization.

As an example of plausible causal evidence from a Mendelian randomization investigation despite only nominal statistical significance, Gill and Burgess investigated a rare genetic variant (minor allele frequency 0.3%) in the *F10* gene[52] that has previously been shown to associate with plasma activated factor X (FXa) levels at a genome-wide level of statistical significance.[53] FXa inhibitors, such as rivaroxaban and apixaban, have been shown to be effective at reducing the risk of venous thromboembolism in randomized trials[54] and have been used in its treatment for more than 10 years. In this analysis, the variant was associated with lower risk of pulmonary embolism ($p = 0.0006$) and deep vein thrombosis ($p = 0.051$); pulmonary embolism and deep vein thrombosis are subtypes of

Table 1. Summary of differences between Mendelian randomization and colocalization

Mendelian randomization	Colocalization
Motivation is to investigate evidence for the causal effect of an exposure on an outcome	Motivation is to understand the relationship between genetic signals at a locus
Asymmetric in the traits: one trait is the exposure, the other is the outcome	Symmetric in the traits: the traits are treated equivalently in the analysis
Can focus on a single genetic region, but often polygenic	Focuses on a single genetic region
Choice of genetic region is driven by association with the exposure	Choice of genetic region is motivated by overlapping signals at a locus
Often one variant per genetic region (and rarely more than a few)	Dense coverage of variants in the genetic region is required
Does not have to include causal variants	Assumes causal variant is measured
Assumes that genetic variants used satisfy instrumental variable assumptions	No assumption about the genetic variants
Output is an estimate that can be interpreted as a hypothesis test statistic	Output is a set of posterior probabilities for different hypotheses (for enumeration colocalization)
Results are typically interpreted through the lens of causality	Results are agnostic to the causal model between the traits
Generally more liberal (higher probability of false positive). Mendelian randomization methods assume that the genetic variant(s) can be interpreted as proxies for intervention on the exposure, and hence even a weak association between the variants and the outcome may be indicative of a causal effect.	Generally more conservative. Enumeration colocalization methods employ sceptical priors in accordance with genome-wide testing practice; they generally require strong statistical evidence of associations with traits to conclude there is colocalization.

venous thromboembolism. The variant was also associated with increased risk of subarachnoid hemorrhage ($p = 0.031$); bleeding is a known adverse effect of FXa inhibition. Due to our previous understanding of this mechanism and its impact in clinical practice, the genetic evidence that FXa inhibition increases the risk of subarachnoid hemorrhage is convincing despite the moderate strength of the statistical association.

The output from a Mendelian randomization analysis is typically interpreted in a causal framework as evidence of whether the exposure has a causal effect on the outcome. However, we underscore that any statistical method that makes causal claims does so on the basis of assumptions. To state that a Mendelian randomization analysis enables the analyst to make a causal claim is a circular argument; a causal conclusion is only possible if the analyst has made sufficient assumptions to justify a causal claim. Authors should therefore be cautious not to overstate any causal claims, particularly from *cis*-Mendelian randomization analyses, as all the evidence in such an analysis comes from a single genetic region.[55] In contrast, colocalization analyses are agnostic to the model relating the traits. Colocalization could be inferred either if trait 1 had a causal effect on trait 2, if trait 2 had a causal effect on trait 1, or if both traits were influenced by a common cause.

Departures from proportionality in the genetic association estimates, referred to in Mendelian randomization as heterogeneity in the variant-specific estimates,[38] are interpreted differently by the two approaches. In Mendelian randomization, if a random-effect analysis method is used, heterogeneity leads to wider confidence intervals for the causal estimate. However, it is still possible that the confidence interval for the causal estimate excludes the null. In contrast, heterogeneity in colocalization is interpreted as evidence against colocalization. For a polygenic Mendelian randomization analysis with a non-zero causal estimate, some degree of heterogeneity may be expected, as variants in different genetic regions that influence the exposure via different mechanisms may have different proportional associations with the outcome. Heterogeneity can also be interpreted as evidence for invalidity of the instrumental variable assumptions for some variants, particularly if heterogeneity is substantial or is attributable to a small number of outlying variants.[56]

A summary of these differences between the two approaches is provided as Table 1.

Conflicting findings from Mendelian randomization and colocalization

Mendelian randomization and colocalization can give results that appear to be in conflict. If the exposure and outcome traits colocalize at a particular locus, then a Mendelian randomization analysis using variants from that locus will generally provide a non-zero estimate. An exception is the implausible case that some genetic predictors of the exposure are positively associated with the outcome and others are negatively associated with the outcome, leading to an overall zero Mendelian randomization estimate. However, this is very unlikely to happen in practice.

More often, a non-zero Mendelian randomization estimate is found without evidence for colocalization. One reason why the methods may provide conflicting answers is that the exposure and outcome have distinct causal variants that are in linkage disequilibrium, meaning that the Mendelian randomization assumptions are violated. In this case, using notation from the coloc method, we might

expect a colocalization analysis to support the $\mathbb{H}_3$ hypothesis, that of distinct causal variants. Alternatively, colocalization might indicate insufficient evidence for association with one or both traits in the given data. In this case, we might expect colocalization to support one of the hypotheses $\mathbb{H}_0$, $\mathbb{H}_1$, or $\mathbb{H}_2$. Having been developed in the GWAS context, where the analyst must consider all other hypotheses that could be proposed across the genome, colocalization typically requires stronger evidence of association to support the $\mathbb{H}_3$ or $\mathbb{H}_4$ hypothesis than is required in a Mendelian randomization analysis to produce a non-zero estimate. We illustrate these scenarios in analyses below.

Finally, colocalization analyses may be difficult to interpret due to an underlying complexity of the genetic region, such as the presence of allelic heterogeneity.[57] Difficulties may arise when one of the traits is complex, and hence it may be affected by multiple biological mechanisms, or for a molecular trait, as it is more likely that several distinct causal variants can be detected. This should be investigated using colocalization methods that can incorporate multiple causal variants.[33,36] There may be instances where there are separate causal variants related to distinct effects of the same gene (either its expression or function) that differentially influence different outcomes. One plausible such example is *GLP1R*, where there are distinct genome-wide significant signals for type 2 diabetes[58] and BMI;[59] a colocalization analysis at this locus provided evidence of distinct causal variants for these traits (probability of $\mathbb{H}_4 < 1\%$). This genetic evidence supports the notion that GLP1R perturbation affects glycemic control and body weight through different mechanisms.[60] Generally speaking, colocalization results can be sensitive to the choice of traits in the analysis, and in particular analyses using mRNA expression can be sensitive to the choice of tissue. In contrast, Mendelian randomization analyses are more sensitive to the choice of genetic variants rather than the choice of the exposure, as Mendelian randomization findings chiefly depend on genetic associations with the outcome.

Perspectives and applications

While both Mendelian randomization and colocalization have been used for a variety of different subject areas, Mendelian randomization has generally been used for diseases that have recognized risk factors, such as cardiometabolic diseases,[61] whereas applications of colocalization have been more common for auto-immune diseases.[39,62] This reflects an attempt to understand pleiotropy in genetic associations with auto-immune diseases.[63] Most exposures used in Mendelian randomization analyses are clinical biomarkers or phenotypic traits, although the approach has been applied to consider other molecular exposures, including mRNA expression and protein levels.[64,65] In contrast, most applications of colocalization have considered molecular traits and/or disease outcomes.

As discussed above, colocalization is increasingly being used as a sensitivity analysis for Mendelian randomization.[66–69] For example, Zheng et al. performed a phenome-wide Mendelian randomization investigation considering circulating levels of various proteins as exposures.[70] Out of the 413 protein-outcome pairs with supporting evidence for causation from Mendelian randomization, 283 (68.5%) were supported by evidence from colocalization, defined as a posterior probability for $\mathbb{H}_4$ above 80%. Out of the 1,002 proteins considered, 153 had multiple conditionally distinct predictors in their relevant gene region. The authors addressed this by first identifying conditionally independent signals using the GCTA-COJO package, and then performing pairwise colocalization analyses for these signals. For a substantial number of the protein-outcome pairs, strong evidence for colocalization was detected only after applying this approach (23 out of 283, 8.1%). These analyses preceded the development of the coloc-SuSiE[33] and CAFEH methods,[36] which facilitate enumeration colocalization analyses with multiple causal variants. While in some cases, failure to colocalize was due to lack of strong associations with the exposure and/or outcome, in other cases, strong evidence was observed supporting the $\mathbb{H}_3$ hypothesis of distinct causal variants.

Illustrative examples: LDL cholesterol, coronary heart disease, and Alzheimer disease

We illustrate these points by performing Mendelian randomization and colocalization analyses using summarized genetic associations with LDL cholesterol estimated in up to 188,577 individuals of European ancestries from the Global Lipid Genetics Consortium (GLGC) 2013 data release,[71] CHD risk in up to 60,801 affected individuals and 123,504 control individuals from the multi-ethnic CARDIoGRAMplusC4D Consortium,[72] and Alzheimer disease in up to 17,008 affected individuals and 37,154 control subjects of European ancestries (discovery phase only) from the International Genomics of Alzheimer's Project (IGAP) consortium.[73] We also consider colocalization using genetic associations with protein levels from plasma of proprotein convertase subtilisin/kexin type 9 (PCSK9) estimated in 35,559 Icelanders.[74]

Genetic associations for 75 variants associated with LDL cholesterol at a genome-wide level of statistical significance ($p < 5 \times 10^{-8}$) in the 2013 GLGC analysis[75] are displayed in Figure 3 (left, CHD; right, Alzheimer disease). For CHD, a polygenic Mendelian randomization analysis based on these variants suggests a causal effect of higher LDL cholesterol on CHD. The random-effects IVW estimate, representing the average association with the outcome for a standard deviation increase in genetically predicted LDL cholesterol, is an odds ratio (OR) of 1.53 (95% confidence interval [CI]: 1.40, 1.67). This is despite a Cochran's Q heterogeneity test statistic, which represents heterogeneity in the variant-specific estimates, of 282.1

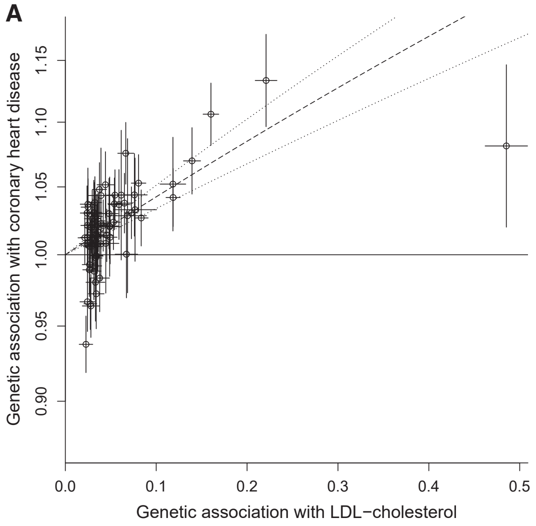

Figure 3. Scatter plots of genetic associations with LDL cholesterol, coronary heart disease, and Alzheimer disease
Genetic associations with LDL cholesterol (horizontal axis, standard deviation units) against genetic associations with (A) coronary heart disease and (B) Alzheimer disease (vertical axis, odds ratios) for 75 genetic variants associated with LDL cholesterol. Error bars represent 95% confidence intervals for the genetic associations; dashed line represents inverse-variance weighted estimate (dotted lines represent 95% confidence intervals for this estimate). In the right-hand plot, variants in the *APOE* gene region are marked with triangles.

($p < 0.001$). However, the majority of points in the scatterplot are distributed around the IVW estimate (Figure 3, left), suggesting that this may be due to heterogeneity in causal estimates rather than pleiotropy. A similar positive estimate of OR 1.32 (95% CI: 1.12, 1.56) was observed from a *cis*-Mendelian randomization restricted to variants in the *PCSK9* gene region,[76] which encodes an established drug target for preventing cardiovascular disease.[77]

For Alzheimer disease, a polygenic Mendelian randomization analysis gives a random-effects IVW estimate of OR 1.27 (95% CI: 1.04, 1.55). However, it is clear from visual inspection of the scatter plot that two variants are outliers (Figure 3, right; outliers are marked as triangles). These variants (rs634869 and rs12525163) are both in the *APOE* gene region, a locus known to be a strong predictor of Alzheimer disease. Excluding these variants from the analysis, the IVW estimate is OR 1.02 (95% CI: 0.93, 1.12), and Cochran's Q heterogeneity test statistic, which represents heterogeneity in the variant-specific estimates, reduces from 516.1 ($p < 0.001$) to 92.9 ($p = 0.06$). This suggests that any Mendelian randomization evidence for a causal effect of LDL cholesterol on Alzheimer disease risk is dependent on the variants in the *APOE* gene region. A *cis*-Mendelian randomization analysis based on these two variants in the *APOE* gene region suggests a positive effect of LDL cholesterol of OR 4.33 (95% CI: 3.56, 5.26) that is not evidenced by the remainder of the genetic variants.

Although polygenic Mendelian randomization analyses excluding variants from the *APOE* gene region have consistently given null results,[78] a previous *cis*-Mendelian randomization analysis for LDL cholesterol and Alzheimer disease based solely on variants in the *PCSK9* gene region gave an inverse estimate of OR 0.69 (95% CI: 0.59, 0.81) per standard deviation increase in genetically predicted LDL cholesterol, suggesting that lowering LDL cholesterol via PCSK9 inhibition may increase risk of Alzheimer disease.[79]

We next perform colocalization analyses with these traits. First, we consider LDL cholesterol and CHD for the genetic region 100 kilobasepairs either side of the *PCSK9* gene region (chr1:55,505,221–55,530,525 on hg19 by Ensembl), We implement colocalization using the *coloc* method of Giambartolomei et al.[24] with priors set at $p_1 = p_2 = 10^{-4}$ and $p_{12} = 10^{-5}$, where p_1 represents the probability of each variant being the causal variant for trait 1, p_2 represents the probability of each variant being the causal variant for trait 2, and p_{12} represents the probability of each variant being the causal variant for both traits. These priors were originally recommended for the analysis of eQTL data;[80] we use them here as they are most commonly employed in applied practice. We find a posterior probability for $\mathbb{H}_4$ of >99.9%, supporting a shared causal variant for these two traits at the locus. Regional association plots show well-defined peaks in the genetic associations for both traits (Figure 4). This suggests that the signals colocalize, which is consistent with LDL cholesterol being the causal risk factor for CHD at this locus.

Colocalization of protein levels of PCSK9 and CHD risk using the coloc method showed convincing but weaker evidence of colocalization (posterior probability for $\mathbb{H}_4$ of 82.4%). Results were sensitive to the choice of prior for p_{12}, as indicated by running the sensitivity() function in the *coloc* package (Figure S1). However, there was some evidence of multiple causal variants for PCSK9, as there were near-independent variants ($r^2 < 0.1$) with low p values.

We attempted to repeat analyses using coloc-SuSiE, which allows for the existence of multiple causal variants. However, any fine-mapping approach using GWAS summary

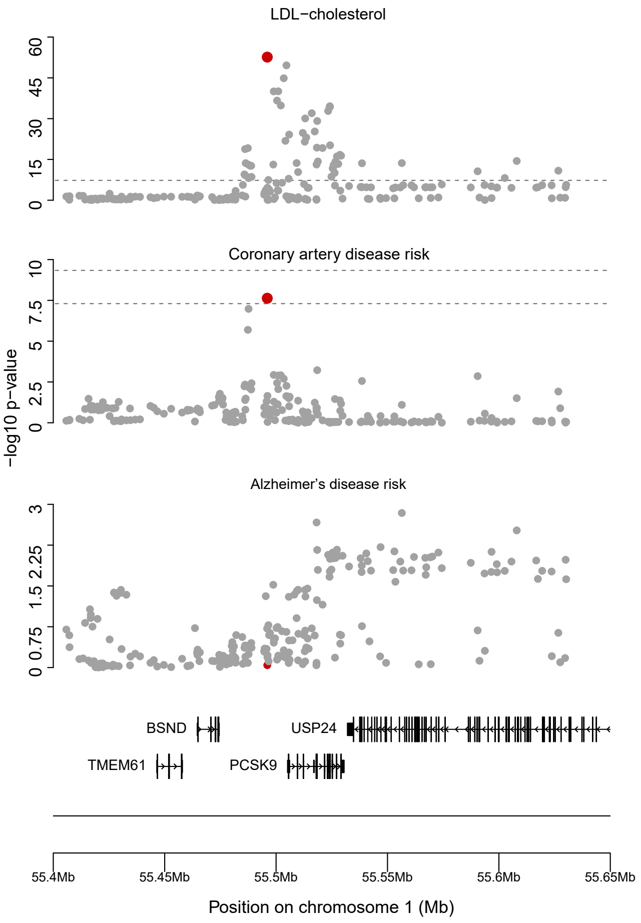

Figure 4. Regional association plots for the *PCSK9* gene region
Genetic associations (negative $\log_{10}$ p values) plotted against chromosome position (megabases, Mb) for variants around the *PCSK9* gene region with LDL cholesterol, coronary heart disease risk, and Alzheimer disease risk. Note the well-defined peak around the lead variant for both LDL cholesterol and coronary heart disease (marked in red), and the absence of a well-defined peak around any lead variant for Alzheimer disease. Colocalization suggests that LDL cholesterol and coronary heart disease have a shared causal variant, which is this lead variant, and no evidence that there is a causal variant for Alzheimer disease at this locus. Figures were made using the karyoploteR package: http://bioconductor.org/packages/release/bioc/html/karyoploteR.html.

statistics requires an estimate of the sample linkage disequilibrium matrix, and the accuracy of inference from these methods is very sensitive to the accuracy of this matrix.[35,81] We were unable to find publicly available data to estimate linkage disequilibrium in an Icelandic population. Instead, we estimated the linkage disequilibrium matrix using 367,703 unrelated participants of European ancestries from UK Biobank, following quality control steps described in Astle et al.[82] SuSiE found evidence of 9 causal variants for PCSK9 (posterior probability > 95%). However, the marginal p values for some of these variants were close to 1, which is implausible. A diagnostic plot indicated disagreement between the genetic associations and the correlation matrix, likely due to different linkage disequilibrium patterns between the Icelandic population and European ancestry individuals from UK Biobank; this is implemented using the kriging_rss() function in the *susieR* package.[35] This highlights a practical limitation of methods for fine-mapping, and by implication colocalization, that allow for multiple causal variants.

Performing colocalization for Alzheimer disease using the same gene region and settings as above, we found a posterior probability of a shared causal variant ($\mathbb{H}_4$) of only 1.0%. Instead, the model favored $\mathbb{H}_1$, with a 97.1% posterior probability of the region containing a causal variant for LDL cholesterol but not Alzheimer disease. This is because none of the variants at the locus were strongly associated with Alzheimer disease ($p > 0.001$ for all variants, Figure 4), and hence the power to detect colocalization was low. This finding represents the greater *a priori* skepticism of the colocalization priors; if the causal variant for LDL cholesterol does affect Alzheimer disease risk, the association does not have sufficient strength in these data to outweigh our prior skepticism. The probability of $\mathbb{H}_4$ divided by the sum of the probabilities of $\mathbb{H}_3$ and $\mathbb{H}_4$ $\left(\frac{P(\mathbb{H}_4)}{P(\mathbb{H}_3)+P(\mathbb{H}_4)}\right)$, which represents the probability of colocalization conditional on the presence of a causal variant for Alzheimer disease, is 34.5%, which again suggests no strong evidence for colocalization, although this calculation is strongly dependent on the choice of prior parameter p_{12}. A visual check of the regional association plot suggests no strong evidence of an association with Alzheimer disease at the locus, with an absence of the well-defined peak that typically characterizes a true genetic association (Figure 4).

Finally, we consider colocalization of LDL cholesterol and Alzheimer disease risk at the genetic region 100 kilobasepairs either side of the *APOE* gene (chr19:45,409,011–45,412,650 on hg19 by Ensembl) where *cis*-Mendelian randomization suggests a causal relationship. Performing colocalization using the same settings gives a posterior probability for $\mathbb{H}_3$ of >99.9%, providing genetic evidence to support separate causal variants underlying the associations at this locus (Figure 5). This suggests that the positive estimate in the polygenic Mendelian randomization analysis including the *APOE* variants arises from violation of the instrumental variable assumptions due to linkage disequilibrium. This emphasizes the importance of checking for outliers in polygenic Mendelian randomization analyses and using colocalization to test the Mendelian randomization assumptions at a particular locus.

Extensions and future directions

There are several extensions to Mendelian randomization, including network Mendelian randomization, which assesses mediation of the causal effect of an exposure via a mediating trait;[83] non-linear Mendelian randomization, which assesses the shape of the causal relationship between an exposure and an outcome;[84] factorial Mendelian randomization, which assesses whether there are

Please cite this article in press as: Zuber et al., Combining evidence from Mendelian randomization and colocalization: Review and comparison of approaches, The American Journal of Human Genetics (2022), https://doi.org/10.1016/j.ajhg.2022.04.001

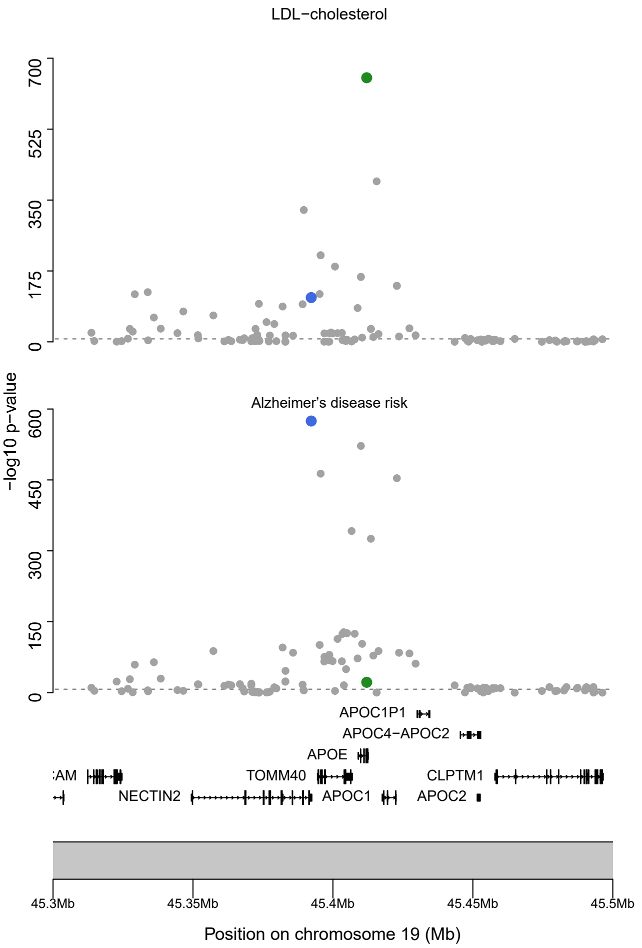

Figure 5. Regional association plots for the *APOE* gene region
Genetic associations (negative $\log_{10}$ p values) plotted against chromosome position (megabases, Mb) for variants around the *APOE* gene region with LDL cholesterol and Alzheimer disease risk. Note the well-defined peak around the lead variant for both traits (marked in green for LDL cholesterol, and blue for Alzheimer disease). However, in this case, colocalization suggests the peaks have distinct causal variants.

interactions between exposures (or interventions) in their effects on an outcome;[85] and bidirectional Mendelian randomization, which assesses the causal effects of the exposure on the outcome and of the outcome on the exposure using different sets of genetic variants.[44] Equally, there are extensions to colocalization, such as methods for colocalization with cross-population data.[86,87] We here focus on an extension to Mendelian randomization that has a parallel in colocalization: the analysis of multiple traits.

Multivariable Mendelian randomization assesses whether genetically predicted levels of multiple exposures are associated with an outcome in a multivariable model.[88] It is typically used in two contexts: first, to assess the effect of an exposure when genetic variants associated with the exposure of interest may have pleiotropic effects on the outcome via other measured risk factors;[89] and secondly, to assess the relative contribution of causal pathways from the exposure to the outcome via other risk factors.[90] Estimates from multivariable Mendelian randomization can be interpreted as the direct effect of an exposure; that is, the component of the causal effect of an exposure that does not pass via other risk factors included in the analysis.[91] While typically multivariable Mendelian randomization analyses are polygenic, as it is necessary to include some genetic variants in the analysis that have relatively stronger associations with each exposure, a recent methodological development considered *cis*-multivariable Mendelian randomization.[92] This approach was applied to disentangle the causal effects of three related proteins associated with variants at the chemokine receptor gene cluster.

The analogous colocalization method is multiple-trait colocalization, which assesses colocalization between several traits in a single analysis. Such methods include multiple-trait colocalization (moloc)[27] and hypothesis prioritization for multi-trait colocalization (HyPrColoc),[28] which both make the single causal variant assumption. While moloc is typically computationally intractable with 5 or more traits, HyPrColoc can be rapidly implemented with hundreds of traits. The CAFEH method extends on these methods by relaxing the single causal variant assumption, thereby allowing different patterns of colocalization to be detected for multiple traits at each causal variant.[36]

Although both extensions consider relationships between multiple traits, the aims of the methods are in some ways opposing. Multivariable Mendelian randomization aims to disentangle related traits, in order to distinguish which is the causal exposure for a given outcome.[93] As such, if suitable genetic variants are available, it can model complex networks of relationships between traits. In contrast, multiple-trait colocalization aims to find clusters of traits with shared genetic predictors.

A potential future direction for Mendelian randomization and colocalization is to consider how the two approaches can be used in an integrative way. Developments in this direction include the Mendelian randomization (MR)-link method, which accounts for potential pleiotropy at a given locus using ridge regression to discount the effect of invalid instruments; where a variant that is considered invalid is one that would lead to proportional colocalization to fail.[94] Another related method is MRLocus, which combines a colocalization step and a Mendelian randomization slope fitting step in a Bayesian hierarchical model, allowing for multiple causal variants and allelic heterogeneity.[95]

Discussion

Mendelian randomization and colocalization have related goals but were developed to serve different scientific communities. This is reflected in their implementation and the interpretation of their results. When considering an exposure with several genetic predictors in different regions, the robustness of Mendelian randomization findings can be

assessed by a range of statistical methods. In such a case, Mendelian randomization is distinct from colocalization, as colocalization only considers associations at a single genetic region. When considering genetic predictors of an exposure in a single region, both Mendelian randomization and colocalization can be performed. However, even though robust methods cannot generally be applied in *cis*-Mendelian randomization investigations, analysts may still require only nominal significance to claim evidence of a causal effect, whereas colocalization typically adopts priors that reflect the GWAS community's high bar for evidence of colocalization. This may be reasonable if the Mendelian randomization analysis is performed using a biologically justified choice of genetic variants to test a specific causal hypothesis, whereas GWASs are typically exploratory "hypothesis-free" investigations, and so correction for multiple testing is essential.

Generally speaking, Mendelian randomization prioritizes the detection of evidence for a causal relationship, whereas colocalization is more conservative. Mendelian randomization simply tests whether there is any average genetic association with the outcome among the genetic predictors of the exposure, whereas colocalization tests for overlap in the genetic variants driving the associations with the traits. As such, colocalization is an important complementary analysis for a *cis*-Mendelian randomization investigation to assess the validity of the instrumental variable assumptions. Without this, *cis*-Mendelian randomization analyses can provide false positive findings similarly to candidate gene studies, which have now largely been abandoned due to providing findings that failed to replicate.[96]

An example of this is found in the contrasting results for LDL cholesterol and coronary heart disease at the *PCSK9* gene region and those for LDL cholesterol and Alzheimer disease at the *APOE* gene region. In the first case, a positive *cis*-Mendelian randomization estimate is strongly supported by colocalization, whereas in the second case the *cis*-Mendelian randomization result is challenged by colocalization, which finds evidence that the traits are influenced by distinct causal variants. The colocalization results are supported by the patterns of association genome-wide, where consistent associations of LDL cholesterol increasing variants with Alzheimer disease are not found.

We therefore strongly recommend that positive *cis*-Mendelian randomization analyses be accompanied by a corresponding colocalization analysis, as is becoming more common in the literature. We acknowledge that this has not always been our own practice over the past years, and will seek to follow this advice more closely in this regard. We note that a negative colocalization finding does not necessarily imply that the target is not valid, but it should prompt the analyst to investigate further why there is a lack of colocalization (for example, whether data sources and the exposure trait have been appropriately selected). If a mitigating reason is not found, this should lessen enthusiasm in the finding, particularly if there is evidence for distinct causal variants and hence separate mechanisms influencing the exposure and outcome. A further note is that this will lead to colocalization analyses that would not otherwise have been performed because of the absence of a strong association with the outcome at the locus. Care should be taken to distinguish findings indicating lack of evidence for association from those where there is strong evidence for distinct causal variants—these scenarios are represented by separate hypotheses in output from the coloc method. While performing colocalization analyses may lead to apparent conflict between results from Mendelian randomization and colocalization, any additional caution arising from this disagreement is often appropriate.

Supplemental information

Supplemental information can be found online at https://doi.org/10.1016/j.ajhg.2022.04.001.

Acknowledgments

S.B. is supported by a Sir Henry Dale Fellowship jointly funded by the Wellcome Trust and the Royal Society (204623/Z/16/Z). C.W. is funded by the Wellcome Trust (WT220788). This research was funded by United Kingdom Research and Innovation Medical Research Council (MC_UU_00002/4 and MC_UU_00002/7), GSK, and MSD, and was supported by the National Institute for Health Research Cambridge Biomedical Research Centre (BRC-1215-20014). The views expressed are those of the authors and not necessarily those of the National Health Service, the National Institute for Health Research or the Department of Health and Social Care. This research has been conducted using the UK Biobank Resource under Application Number 7439.

Declaration of interests

D.G. is a part-time employee of Novo Nordisk. I.M. and C.W. are wholly or partially funded by a grant from GSK and MSD. The other authors have no relevant conflict of interest to declare.

References

1. Freedman, M.L., Monteiro, A.N.A., Gayther, S.A., Coetzee, G.A., Risch, A., Plass, C., Casey, G., De Biasi, M., Carlson, C., Duggan, D., et al. (2011). Principles for the post-GWAS functional characterization of cancer risk loci. Nat. Genet. *43*, 513–518. https://doi.org/10.1038/ng.840.
2. Gallagher, M.D., and Chen-Plotkin, A.S. (2018). The post-GWAS era: from association to function. Am. J. Hum. Genet. *102*, 717–730. https://doi.org/10.1016/j.ajhg.2018.04.002.
3. Sheiner, L.B., and Rubin, D.B. (1995). Intention-to-treat analysis and the goals of clinical trials. Clin. Pharmacol. Ther. *57*, 6–15. https://doi.org/10.1016/0009-9236(95)90260-0.
4. Hingorani, A., and Humphries, S. (2005). Nature's randomised trials. The Lancet *366*, 1906–1908. https://doi.org/10.1016/s0140-6736(05)67767-7.

5. Thanassoulis, G., and O'Donnell, C.J. (2009). Mendelian randomization: nature's randomized trial in the post-genome era. J. Am. Med. Assoc. *301*, 2386–2388. https://doi.org/10.1001/jama.2009.812.
6. Didelez, V., and Sheehan, N. (2007). Mendelian randomization as an instrumental variable approach to causal inference. Stat. Methods Med. Res. *16*, 309–330. https://doi.org/10.1177/0962280206077743.
7. Labrecque, J.A., and Swanson, S.A. (2018). Understanding the assumptions underlying instrumental variable analyses: a brief review of falsification strategies and related tools. Curr. Epidemiol. Rep. *5*, 214–220. https://doi.org/10.1007/s40471-018-0152-1.
8. Davey Smith, G. (2011). Random allocation in observational data: how small but robust effects could facilitate hypothesis-free causal inference. Epidemiology *22*, 460–463. discussion 467-8–463. https://doi.org/10.1097/ede.0b013e31821d0426.
9. Smith, G.D., Lawlor, D.A., Harbord, R., Timpson, N., Day, I., and Ebrahim, S. (2007). Clustered environments and randomized genes: a fundamental distinction between conventional and genetic epidemiology. PLoS Med. *4*, e352. https://doi.org/10.1371/journal.pmed.0040352.
10. Taylor, M., Tansey, K.E., Lawlor, D.A., Bowden, J., Evans, D.M., Davey, S.G., and Timpson, N.J. (2017). Testing the principles of Mendelian randomization: Opportunities and complications on a genomewide scale. Preprint at bioRxiv. https://doi.org/10.1101/124362.
11. Burgess, S., Swanson, S.A., and Labrecque, J.A. (2021). Are Mendelian randomization investigations immune from bias due to reverse causation? Eur. J. Epidemiol. *36*, 253–257. https://doi.org/10.1007/s10654-021-00726-8.
12. Lawlor, D.A., Harbord, R.M., Sterne, J.A.C., Timpson, N., and Davey Smith, G. (2008). Mendelian randomization: using genes as instruments for making causal inferences in epidemiology. Stat. Med. *27*, 1133–1163. https://doi.org/10.1002/sim.3034.
13. Burgess, S., Butterworth, A.S., and Thompson, S.G. (2013). Mendelian randomization analysis with multiple genetic variants using summarized data. Genet. Epidemiol. *37*, 658–665. https://doi.org/10.1002/gepi.21758.
14. Pierce, B.L., and Burgess, S. (2013). Efficient design for Mendelian randomization studies: subsample and 2-sample instrumental variable estimators. Am. J. Epidemiol. *178*, 1177–1184. https://doi.org/10.1093/aje/kwt084.
15. Burgess, S., Scott, R.A., Timpson, N.J., Davey Smith, G., Thompson, S.G.; and EPIC-InterAct Consortium (2015). Using published data in Mendelian randomization: a blueprint for efficient identification of causal risk factors. Eur. J. Epidemiol. *30*, 543–552. https://doi.org/10.1007/s10654-015-0011-z.
16. Burgess, S., Davey Smith, G., Davies, N.M., Dudbridge, F., Gill, D., Glymour, M.M., Hartwig, F.P., Holmes, M.V., Minelli, C., Relton, C.L., et al. (2019). Guidelines for performing Mendelian randomization investigations. Wellcome Open Res. *4*, 186. https://doi.org/10.12688/wellcomeopenres.15555.2.
17. Slob, E.A.W., and Burgess, S. (2020). A comparison of robust Mendelian randomization methods using summary data. Genet. Epidemiol. *44*, 313–329. https://doi.org/10.1002/gepi.22295.
18. van Kippersluis, H., and Rietveld, C.A. (2018). Pleiotropy-robust mendelian randomization. Int. J. Epidemiol. *47*, 1279–1288. https://doi.org/10.1093/ije/dyx002.
19. Spiller, W., Slichter, D., Bowden, J., and Davey Smith, G. (2019). Detecting and correcting for bias in Mendelian randomization analyses using gene-by-environment interactions. Int. J. Epidemiol. *48*, 702–712. https://doi.org/10.1093/ije/dyy204.
20. Schmidt, A.F., Finan, C., Gordillo-Marañón, M., Asselbergs, F.W., Freitag, D.F., Patel, R.S., Tyl, B., Chopade, S., Faraway, R., Zwierzyna, M., et al. (2020). Genetic drug target validation using Mendelian randomisation. Nat. Commun. *11*, 3255. https://doi.org/10.1038/s41467-020-16969-0.
21. Gill, D., Georgakis, M.K., Walker, V.M., Schmidt, A.F., Gkatzionis, A., Freitag, D.F., Finan, C., Hingorani, A.D., Howson, J.M.M., Burgess, S., et al. (2021). Mendelian randomization for studying the effects of perturbing drug targets. Wellcome Open Res. *6*, 16. https://doi.org/10.12688/wellcomeopenres.16544.2.
22. Plagnol, V., Smyth, D.J., Todd, J.A., and Clayton, D.G. (2009). Statistical independence of the colocalized association signals for type 1 diabetes and *RPS26* gene expression on chromosome 12q13. Biostatistics *10*, 327–334. https://doi.org/10.1093/biostatistics/kxn039.
23. Wallace, C. (2013). Statistical testing of shared genetic control for potentially related traits. Genet. Epidemiol. *37*, 802–813. https://doi.org/10.1002/gepi.21765.
24. Giambartolomei, C., Vukcevic, D., Schadt, E.E., Franke, L., Hingorani, A.D., Wallace, C., and Plagnol, V. (2014). Bayesian test for colocalisation between pairs of genetic association studies using summary statistics. PLoS Genet. *10*, e1004.383. https://doi.org/10.1371/journal.pgen.1004383.
25. Claussnitzer, M., Dankel, S.N., Kim, K.H., Quon, G., Meuleman, W., Haugen, C., Glunk, V., Sousa, I.S., Beaudry, J.L., Puviindran, V., et al. (2015). *FTO* obesity variant circuitry and adipocyte browning in humans. New Engl. J. Med. *373*, 895–907. https://doi.org/10.1056/NEJMoa1502214.
26. Chen, W., Larrabee, B.R., Ovsyannikova, I.G., Kennedy, R.B., Haralambieva, I.H., Poland, G.A., and Schaid, D.J. (2015). Fine mapping causal variants with an approximate Bayesian method using marginal test statistics. Genetics *200*, 719–736. https://doi.org/10.1534/genetics.115.176107.
27. Giambartolomei, C., Zhenli Liu, J., Zhang, W., Hauberg, M., Shi, H., Boocock, J., Pickrell, J., Jaffe, A.E., Consortium, C., Pasaniuc, B., et al. (2018). A Bayesian framework for multiple trait colocalization from summary association statistics. Bioinformatics *34*, 2538–2545. https://doi.org/10.1093/bioinformatics/bty147.
28. Foley, C.N., Staley, J.R., Breen, P.G., Sun, B.B., Kirk, P.D.W., Burgess, S., and Howson, J.M.M. (2021). A fast and efficient colocalization algorithm for identifying shared genetic risk factors across multiple traits. Nat. Commun. *12*, 764. https://doi.org/10.1038/s41467-020-20885-8.
29. Wakefield, J. (2009). Bayes factors for genome-wide association studies: comparison with *P*-values. Genet. Epidemiol. *33*, 79–86. https://doi.org/10.1002/gepi.20359.
30. Zhu, Z., Zhang, F., Hu, H., Bakshi, A., Robinson, M.R., Powell, J.E., Montgomery, G.W., Goddard, M.E., Wray, N.R., Visscher, P.M., et al. (2016). Integration of summary data from GWAS and eQTL studies predicts complex trait gene targets. Nat. Genet. *48*, 481–487. https://doi.org/10.1038/ng.3538.
31. Hormozdiari, F., Van De Bunt, M., Segre, A.V., Li, X., Joo, J.W.J., Bilow, M., Sul, J.H., Sankararaman, S., Pasaniuc, B., and Eskin, E. (2016). Colocalization of GWAS and eQTL signals detects target genes. Am. J. Hum. Genet. *99*, 1245–1260. https://doi.org/10.1016/j.ajhg.2016.10.003.

32. Asimit, J.L., Rainbow, D.B., Fortune, M.D., Grinberg, N.F., Wicker, L.S., and Wallace, C. (2019). Stochastic search and joint fine-mapping increases accuracy and identifies previously unreported associations in immune-mediated diseases. Nat. Commun. *10*, 3216. https://doi.org/10.1038/s41467-019-11271-0.
33. Wallace, C. (2021). A more accurate method for colocalisation analysis allowing for multiple causal variants. PLoS Genet. *17*, e1009.440. https://doi.org/10.1371/journal.pgen.1009440.
34. Wang, G., Sarkar, A., Carbonetto, P., and Stephens, M. (2020). A simple new approach to variable selection in regression, with application to genetic fine mapping. J. R. Stat. Soc. Ser. B Stat. Methodol. *82*, 1273–1300. https://doi.org/10.1111/rssb.12388.
35. Zou, Y., Carbonetto, P., Wang, G., and Stephens, M. (2021). Fine-mapping from summary data with the "sum of single effects" model. Preprint at bioRxiv. https://doi.org/10.1101/2021.11.03.467167.
36. Arvanitis, M., Tayeb, K., Strober, B.J., and Battle, A. (2022). Redefining tissue specificity of genetic regulation of gene expression in the presence of allelic heterogeneity. Am. J. Hum. Genet. *109*, 223–239. https://doi.org/10.1016/j.ajhg.2022.01.002.
37. Burgess, S., Dudbridge, F., and Thompson, S.G. (2016). Combining information on multiple instrumental variables in Mendelian randomization: comparison of allele score and summarized data methods. Stat. Med. *35*, 1880–1906. https://doi.org/10.1002/sim.6835.
38. Bowden, J., Hemani, G., and Davey Smith, G. (2018). Detecting individual and global horizontal pleiotropy in Mendelian randomization – a job for the humble heterogeneity statistic? Am. J. Epidemiol. *187*, 2681–2685. https://doi.org/10.1093/aje/kwy185.
39. Wallace, C., Rotival, M., Cooper, J.D., Rice, C.M., Yang, J.H., McNeill, M., Smyth, D.J., Niblett, D., Cambien, F., Cardiogenics Consortium, et al. (2012). Statistical colocalization of monocyte gene expression and genetic risk variants for type 1 diabetes. Hum. Mol. Genet. *21*, 2815–2824. https://doi.org/10.1093/hmg/dds098.
40. Greco M, F.D., Minelli, C., Sheehan, N.A., and Thompson, J.R. (2015). Detecting pleiotropy in Mendelian randomisation studies with summary data and a continuous outcome. Stat. Med. *34*, 2926–2940. https://doi.org/10.1002/sim.6522.
41. Taylor, A.E., Davies, N.M., Ware, J.J., VanderWeele, T., Smith, G.D., and Munafò, M.R. (2014). Mendelian randomization in health research: using appropriate genetic variants and avoiding biased estimates. Econ. Hum. Biol. *13*, 99–106. https://doi.org/10.1016/j.ehb.2013.12.002.
42. Burgess, S., Thompson, S.G.; and CRP CHD Genetics Collaboration (2011). Avoiding bias from weak instruments in Mendelian randomization studies. Int. J. Epidemiol. *40*, 755–764. https://doi.org/10.1093/ije/dyr036.
43. Burgess, S., Davies, N.M., and Thompson, S.G. (2016). Bias due to participant overlap in two-sample Mendelian randomization. Genet. Epidemiol. *40*, 597–608. https://doi.org/10.1002/gepi.21998.
44. Hemani, G., Tilling, K., and Davey Smith, G. (2017). Orienting the causal relationship between imprecisely measured traits using GWAS summary data. PLoS Genet. *13*, e1007.081. https://doi.org/10.1371/journal.pgen.1007081.
45. Burgess, S., O'Donnell, C.J., and Gill, D. (2021). Expressing results from a Mendelian randomization analysis: separating results from inferences. JAMA Cardiol. *6*, 7–8. https://doi.org/10.1001/jamacardio.2020.4317.
46. Hernán, M.A., and Robins, J.M. (2006). Instruments for causal inference: an epidemiologist's dream? Epidemiology *17*, 360–372. https://doi.org/10.1097/01.ede.0000222409.00878.37.
47. Burgess, S., Butterworth, A., Malarstig, A., and Thompson, S.G. (2012). Use of Mendelian randomisation to assess potential benefit of clinical intervention. Br. Med. J. *345*, e7325. https://doi.org/10.1136/bmj.e7325.
48. Ference, B.A. (2018). How to use Mendelian randomization to anticipate the results of randomized trials. Eur. Heart J. *39*, 360–362. https://doi.org/10.1093/eurheartj/ehx462.
49. Nitsch, D., Molokhia, M., Smeeth, L., DeStavola, B.L., Whittaker, J.C., and Leon, D.A. (2006). Limits to causal inference based on Mendelian randomization: a comparison with randomized controlled trials. Am. J. Epidemiol. *163*, 397–403. https://doi.org/10.1093/aje/kwj062.
50. VanderWeele, T.J., Tchetgen Tchetgen, E.J., Cornelis, M., and Kraft, P. (2014). Methodological challenges in Mendelian randomization. Epidemiology *25*, 427–435. https://doi.org/10.1097/ede.0000000000000081.
51. Lawlor, D.A., Tilling, K., and Davey Smith, G. (2016). Triangulation in aetiological epidemiology. Int. J. Epidemiol. *45*, 1866–1886. https://doi.org/10.1093/ije/dyw314.
52. Gill, D., and Burgess, S. (2020). Use of a genetic variant related to circulating FXa (Activated Factor X) levels to proxy the effect of FXa inhibition on cardiovascular outcomes. Circ. Genomic Precision Med. *13*, 551–553. https://doi.org/10.1161/circgen.120.003061.
53. Sun, B.B., Maranville, J.C., Peters, J.E., Stacey, D., Staley, J.R., Blackshaw, J., Burgess, S., Jiang, T., Paige, E., Surendran, P., et al. (2018). Genomic atlas of the human plasma proteome. Nature *558*, 73–79. https://doi.org/10.1038/s41586-018-0175-2.
54. Carrier, M., Abou-Nassar, K., Mallick, R., Tagalakis, V., Shivakumar, S., Schattner, A., Kuruvilla, P., Hill, D., Spadafora, S., Marquis, K., et al. (2019). Apixaban to prevent venous thromboembolism in patients with cancer. New Engl. J. Med. *380*, 711–719. https://doi.org/10.1056/NEJMoa1814468.
55. Davey Smith, G., Davies, N.M., Dimou, N., Egger, M., Gallo, V., Golub, R., Higgins, J.P., Langenberg, C., Loder, E.W., Richards, J.B., et al. (2019). STROBE-MR: Guidelines for strengthening the reporting of Mendelian randomization studies. PeerJ Preprints *7*, e27. https://doi.org/10.7287/peerj.preprints.27857v1.
56. Verbanck, M., Chen, C.Y., Neale, B., and Do, R. (2018). Detection of widespread horizontal pleiotropy in causal relationships inferred from Mendelian randomization between complex traits and diseases. Nat. Genet. *50*, 693–698. https://doi.org/10.1038/s41588-018-0099-7.
57. Hormozdiari, F., Zhu, A., Kichaev, G., Ju, C.J.T., Segre, A.V., Joo, J.W.J., Won, H., Sankararaman, S., Pasaniuc, B., Shifman, S., et al. (2017). Widespread allelic heterogeneity in complex traits. Am. J. Hum. Genet. *100*, 789–802. https://doi.org/10.1016/j.ajhg.2017.04.005.
58. Vujkovic, M., Keaton, J.M., Lynch, J.A., Miller, D.R., Zhou, J., Tcheandjieu, C., Huffman, J.E., Assimes, T.L., Lorenz, K., Zhu, X., et al. (2020). Discovery of 318 new risk loci for type 2 diabetes and related vascular outcomes among 1.4 million

Please cite this article in press as: Zuber et al., Combining evidence from Mendelian randomization and colocalization: Review and comparison of approaches, The American Journal of Human Genetics (2022), https://doi.org/10.1016/j.ajhg.2022.04.001

participants in a multi-ancestry meta-analysis. Nat. Genet. *52*, 680–691. https://doi.org/10.1038/s41588-020-0637-y.
59. Pulit, S.L., Stoneman, C., Morris, A.P., Wood, A.R., Glastonbury, C.A., Tyrrell, J., Yengo, L., Ferreira, T., Marouli, E., Ji, Y., et al. (2019). Meta-analysis of genome-wide association studies for body fat distribution in 694 649 individuals of European ancestry. Hum. Mol. Genet. *28*, 166–174. https://doi.org/10.1093/hmg/ddy327.
60. Wilding, J.P., Batterham, R.L., Calanna, S., Davies, M., Van Gaal, L.F., Lingvay, I., McGowan, B.M., Rosenstock, J., Tran, M.T., Wadden, T.A., et al. (2021). Once-weekly semaglutide in adults with overweight or obesity. New Engl. J. Med. *384*, 989–1002. https://doi.org/10.1056/NEJMoa2032183.
61. Boef, A.G.C., Dekkers, O.M., and Le Cessie, S. (2015). Mendelian randomization studies: a review of the approaches used and the quality of reporting. Int. J. Epidemiol. *44*, 496–511. https://doi.org/10.1093/ije/dyv071.
62. Fortune, M.D., Guo, H., Burren, O., Schofield, E., Walker, N.M., Ban, M., Sawcer, S.J., Bowes, J., Worthington, J., Barton, A., et al. (2015). Statistical colocalization of genetic risk variants for related autoimmune diseases in the context of common controls. Nat. Genet. *47*, 839–846. https://doi.org/10.1038/ng.3330.
63. Ellinghaus, D., Jostins, L., Spain, S.L., Cortes, A., Bethune, J., Han, B., Park, Y.R., Raychaudhuri, S., Pouget, J.G., Hübenthal, M., et al. (2016). Analysis of five chronic inflammatory diseases identifies 27 new associations and highlights disease-specific patterns at shared loci. Nat. Genet. *48*, 510–518. https://doi.org/10.1038/ng.3528.
64. Relton, C.L., and Davey Smith, G. (2012). Two-step epigenetic Mendelian randomization: a strategy for establishing the causal role of epigenetic processes in pathways to disease. Int. J. Epidemiol. *41*, 161–176. https://doi.org/10.1093/ije/dyr233.
65. Neumeyer, S., Hemani, G., and Zeggini, E. (2020). Strengthening causal inference for complex disease using molecular quantitative trait loci. Trends Mol. Med. *26*, 232–241. https://doi.org/10.1016/j.molmed.2019.10.004.
66. McGowan, L.M., Davey Smith, G., Gaunt, T.R., and Richardson, T.G. (2019). Integrating mendelian randomization and multiple-trait colocalization to uncover cell-specific inflammatory drivers of autoimmune and atopic disease. Hum. Mol. Genet. *28*, 3293–3300. https://doi.org/10.1093/hmg/ddz155.
67. Karhunen, V., Daghlas, I., Zuber, V., Vujkovic, M., Olsen, A.K., Knudsen, L.B., Haynes, W.G., Howson, J.M.M., and Gill, D. (2021). Leveraging human genetic data to investigate the cardiometabolic effects of glucose-dependent insulinotropic polypeptide signalling. Diabetologia *64*, 2773–2778. https://doi.org/10.1007/s00125-021-05564-7.
68. Baird, D.A., Liu, J.Z., Zheng, J., Sieberts, S.K., Perumal, T., Elsworth, B., Richardson, T.G., Chen, C.Y., Carrasquillo, M.M., Allen, M., et al. (2021). Identifying drug targets for neurological and psychiatric disease via genetics and the brain transcriptome. PLoS Genet. *17*, e1009.224. https://doi.org/10.1371/journal.pgen.1009224.
69. Bouras, E., Karhunen, V., Gill, D., Huang, J., Haycock, P.C., Gunter, M.J., Johansson, M., Brennan, P., Key, T., Lewis, S.J., et al. (2022). Circulating inflammatory cytokines and risk of five cancers: a Mendelian randomization analysis. BMC Med. *20*, 3. https://doi.org/10.1186/s12916-021-02193-0.
70. Zheng, J., Haberland, V., Baird, D., Walker, V., Haycock, P.C., Hurle, M.R., Richardson, T.G., Erola, P., Liu, Y., Luo, S., et al. (2020). Phenome-wide Mendelian randomization mapping the influence of the plasma proteome on complex diseases. Nat. Genet. *52*, 1122–1131. https://doi.org/10.1038/s41588-020-0682-6.
71. The Global Lipids Genetics Consortium (2013). Discovery and refinement of loci associated with lipid levels. Nat. Genet. *45*, 1274–1283. https://doi.org/10.1038/ng.2797.
72. Nikpay, M., Goel, A., Won, H.H., Hall, L.M., Willenborg, C., Kanoni, S., Saleheen, D., Kyriakou, T., Nelson, C.P., Hopewell, J.C., et al. (2015). A comprehensive 1000 Genomes-based genome-wide association meta-analysis of coronary artery disease. Nat. Genet. *47*, 1121–1130. https://doi.org/10.1038/ng.3396.
73. Lambert, J.C., Ibrahim-Verbaas, C.A., Harold, D., Naj, A.C., Sims, R., Bellenguez, C., DeStafano, A.L., DeStefano, A.L., Bis, J.C., Grenier-Boley, B., et al. (2013). Meta-analysis of 74,046 individuals identifies 11 new susceptibility loci for Alzheimer's disease. Nat. Genet. *45*, 1452–1458. https://doi.org/10.1038/ng.2802.
74. Ferkingstad, E., Sulem, P., Atlason, B.A., Sveinbjornsson, G., Magnusson, M.I., Styrmisdottir, E.L., Gunnarsdottir, K., Helgason, A., Oddsson, A., Halldorsson, B.V., et al. (2021). Large-scale integration of the plasma proteome with genetics and disease. Nat. Genet. *53*, 1712–1721. https://doi.org/10.1038/s41588-021-00978-w.
75. Do, R., Willer, C.J., Schmidt, E.M., Sengupta, S., Gao, C., Peloso, G.M., Gustafsson, S., Kanoni, S., Ganna, A., Chen, J., et al. (2013). Common variants associated with plasma triglycerides and risk for coronary artery disease. Nat. Genet. *45*, 1345–1352. https://doi.org/10.1038/ng.2795.
76. Allara, E., Morani, G., Carter, P., Gkatzionis, A., Zuber, V., Foley, C.N., Rees, J.M.B., Mason, A.M., Bell, S., Gill, D., et al. (2019). Genetic determinants of lipids and cardiovascular disease outcomes: a wide-angled Mendelian randomization investigation. Circ. Genomic Precision Med. *12*, e002.711. https://doi.org/10.1161/circgen.119.002711.
77. Sabatine, M.S., Giugliano, R.P., Keech, A.C., Honarpour, N., Wiviott, S.D., Murphy, S.A., Kuder, J.F., Wang, H., Liu, T., Wasserman, S.M., et al. (2017). Evolocumab and clinical outcomes in patients with cardiovascular disease. New Engl. J. Med. *376*, 1713–1722. https://doi.org/10.1056/NEJMoa1615664.
78. Benn, M., Nordestgaard, B.G., Frikke-Schmidt, R., and Tybjærg-Hansen, A. (2017). Low LDL cholesterol, *PCSK9* and *HMGCR* genetic variation, and risk of Alzheimer's disease and Parkinson's disease: mendelian randomisation study. Br. Med. J. *357*, j1648. https://doi.org/10.1136/bmj.j1648.
79. Williams, D.M., Finan, C., Schmidt, A.F., Burgess, S., and Hingorani, A.D. (2020). Lipid lowering and Alzheimer disease risk: a Mendelian randomization study. Ann. Neurol. *87*, 30–39. https://doi.org/10.1002/ana.25642.
80. Wallace, C. (2020). Eliciting priors and relaxing the single causal variant assumption in colocalisation analyses. PLoS Genet. *16*, e1008.720. https://doi.org/10.1371/journal.pgen.1008720.
81. Benner, C., Havulinna, A.S., Järvelin, M.R., Salomaa, V., Ripatti, S., and Pirinen, M. (2017). Prospects of fine-mapping trait-associated genomic regions by using summary statistics from genome-wide association studies. Am. J. Hum. Genet. *101*, 539–551. https://doi.org/10.1016/j.ajhg.2017.08.012.
82. Astle, W.J., Elding, H., Jiang, T., Allen, D., Ruklisa, D., Mann, A.L., Mead, D., Bouman, H., Riveros-Mckay, F., Kostadima,

M.A., et al. (2016). The allelic landscape of human blood cell trait variation and links to common complex disease. Cell *167*, 1415–1429.e19. https://doi.org/10.1016/j.cell.2016.10.042.

83. Burgess, S., Daniel, R.M., Butterworth, A.S., Thompson, S.G.; and EPIC-InterAct Consortium (2015). Network Mendelian randomization: using genetic variants as instrumental variables to investigate mediation in causal pathways. Int. J. Epidemiol. *44*, 484–495. https://doi.org/10.1093/ije/dyu176.
84. Burgess, S., Davies, N.M., Thompson, S.G.; and EPIC-InterAct Consortium (2014). Instrumental variable analysis with a nonlinear exposure–outcome relationship. Epidemiology *25*, 877–885. https://doi.org/10.1097/ede.0000000000000161.
85. Rees, J.M.B., Foley, C.N., and Burgess, S. (2020). Factorial Mendelian randomization: using genetic variants to assess interactions. Int. J. Epidemiol. *49*, 1147–1158. https://doi.org/10.1093/ije/dyz161.
86. Kuchenbaecker, K., Telkar, N., Reiker, T., Walters, R.G., Lin, K., Eriksson, A., Gurdasani, D., Gilly, A., Southam, L., Tsafantakis, E., et al. (2019). The transferability of lipid loci across African, Asian and European cohorts. Nat. Commun. *10*, 4330. https://doi.org/10.1038/s41467-019-12026-7.
87. LaPierre, N., Taraszka, K., Huang, H., He, R., Hormozdiari, F., and Eskin, E. (2021). Identifying causal variants by fine mapping across multiple studies. PLOS Genet. *17*, e1009.733. https://doi.org/10.1371/journal.pgen.1009733.
88. Sanderson, E., Davey Smith, G., Windmeijer, F., and Bowden, J. (2019). An examination of multivariable Mendelian randomization in the single sample and two-sample summary data settings. Int. J. Epidemiol. *48*, 713–727. https://doi.org/10.1093/ije/dyy262.
89. Burgess, S., and Thompson, S.G. (2015). Multivariable Mendelian randomization: the use of pleiotropic genetic variants to estimate causal effects. Am. J. Epidemiol. *181*, 251–260. https://doi.org/10.1093/aje/kwu283.
90. Carter, A.R., Sanderson, E., Hammerton, G., Richmond, R.C., Davey Smith, G., Heron, J., Taylor, A.E., Davies, N.M., and Howe, L.D. (2021). Mendelian randomisation for mediation analysis: current methods and challenges for implementation. Eur. J. Epidemiol. *36*, 465–478. https://doi.org/10.1007/s10654-021-00757-1.
91. Burgess, S., Thompson, D.J., Rees, J.M.B., Day, F.R., Perry, J.R., and Ong, K.K. (2017). Dissecting causal pathways using Mendelian randomization with summarized genetic data: application to age at menarche and risk of breast cancer. Genetics *207*, 481–487. https://doi.org/10.1534/genetics.117.300191.
92. Batool, F., Patel, A., Gill, D., and Burgess, S. (2021). Disentangling the effects of traits with shared clustered genetic predictors using multivariable Mendelian randomization. Preprint at arXiv. https://doi.org/10.48550/arXiv.2109.12361.
93. Zuber, V., Colijn, J.M., Klaver, C., and Burgess, S. (2020). Selecting likely causal risk factors from high-throughput experiments using multivariable Mendelian randomization. Nat. Commun. *11*, 29. https://doi.org/10.1038/s41467-019-13870-3.
94. van der Graaf, A., Claringbould, A., Rimbert, A., Westra, H.J., Westra, H.J., Li, Y., and Wijmenga, C. (2020). Mendelian randomization while jointly modeling *cis* genetics identifies causal relationships between gene expression and lipids. Nat. Commun. *11*, 4930. https://doi.org/10.1038/s41467-020-18716-x.
95. Zhu, A., Matoba, N., Wilson, E.P., Tapia, A.L., Li, Y., Ibrahim, J.G., Stein, J.L., and Love, M.I. (2021). MRLocus: identifying causal genes mediating a trait through Bayesian estimation of allelic heterogeneity. PLoS Genet. *17*, e1009.455. https://doi.org/10.1371/journal.pgen.1009455.
96. Duncan, L.E., Ostacher, M., and Ballon, J. (2019). How genome-wide association studies (GWAS) made traditional candidate gene studies obsolete. Neuropsychopharmacology *44*, 1518–1523. https://doi.org/10.1038/s41386-019-0389-5.

The American Journal of Human Genetics, Volume *109*

Supplemental information

Combining evidence from Mendelian randomization and colocalization: Review and comparison of approaches

Verena Zuber, Nastasiya F. Grinberg, Dipender Gill, Ichcha Manipur, Eric A.W. Slob, Ashish Patel, Chris Wallace, and Stephen Burgess

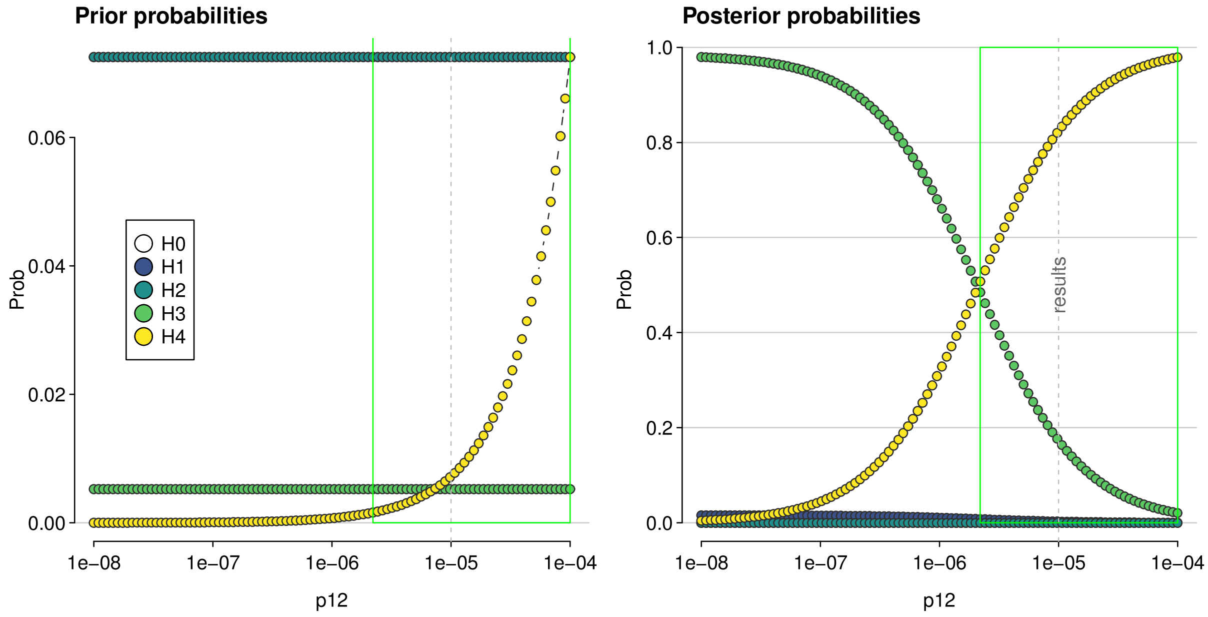

Figure S1: Sensitivity analysis varying the prior p_{12}, which represents the prior probability that any single variant is a causal variant for both traits. The left panel displays the prior probabilities of the hypotheses, the right panel displays the posterior probabilities. For low values of p_{12}, the method prefers the $\mathbb{H}_3$ hypothesis (distinct causal variants), whereas for greater values of p_{12}, the method prefers the $\mathbb{H}_4$ hypothesis (shared causal variant).
